# Knowledge and use of breast self-examination and mammogram among women of reproductive age in Oyo State Secretariat, Ibadan, Oyo State, Nigeria

**DOI:** 10.18332/ejm/105858

**Published:** 2019-04-09

**Authors:** Beatrice Ohaeri, Mosopesoluwa Aderigbigbe

**Affiliations:** 1 University of Ibadan, Ibadan, Nigeria

**Keywords:** knowledge, breast self-examination, mammogram, women, reproductive age

## Abstract

**INTRODUCTION:**

Breast cancer among women is an increasing public health issue. Early detection is the backbone of treatment thus reducing late presentation, morbidity and mortality. In this study, we explored knowledge and use of breast cancer screening among women of reproductive age in Oyo State Secretariat, Ibadan.

**METHODS:**

Descriptive cross-sectional research design was employed to gather data from 204 female workers who consented through purposive sampling technique. A validated semi-structured questionnaire with reliability coefficient of 0.75 was used. Ethical protocols were duly followed. Data were analysed using frequencies, percentages and chi-squared tests at significant level of 0.05 using the Statistical Package for Social Science version 20.

**RESULTS:**

Most of the respondents (82.2%) had adequate knowledge of breast self-examination (BSE) and mammogram, but there was a relatively lower level of utilization of these screening measures. Also, there were significant associations between knowledge, level of education and use of BSE and mammogram (p<0.05).

**CONCLUSIONS:**

In addition to educating women on BSE and mammogram, continuous efforts should be made through workshops and various media to emphasize the significance of practice. This could increase the use of the screening measures leading to a reduction in morbidity and mortality.

## INTRODUCTION

Breast cancer is the most frequent cancer that occurs among women worldwide, and the second leading cause of death, claiming the lives of thousands of women each year and affecting countries at all levels of development^1^. About 1.67 million new cases were diagnosed and 0.522 million deaths from breast cancer occurred in 2012^1^. Although breast cancer is rare in men, it has been estimated that 2470 men were diagnosed with breast cancer worldwide and approximately 460 die each year^2^. The prevalence is rising in most countries and is estimated to rise further over the next 20 years despite current efforts to prevent the disease^3^.The increased incidence is not surprising since there has been, in most countries, an increase in the number of women with major breast cancer risk factors. The risk factors for breast cancer include advancing age^4^, family history of breast cancer, early menarche, late menopause and the use of hormonal replacement therapy (HRT) with combined estrogen and progesterone^5^, late age of first pregnancy, few pregnancies, and short or no periods of breastfeeding. Other risk factors that could be implicated in the burden of breast cancer are obesity, alcohol consumption, and inactivity^6^.

The control of breast cancer in most developing countries including Nigeria and Cameroon is under the auspices of national control programs promoted by the World Health Organization and this involves educating and screening young women for clinical manifestations of breast cancer^6^. The earlier breast cancer is detected, the better the effectiveness of the treatment and the likelihood of survival. Breast cancer screening methods include BSE, Clinical Breast examination (CBE) and mammography, and these are usually done in combination^7^. Among these methods, mammography is the only one that has been proven to be effective, but the method is only cost-effective and feasible in countries with good health infrastructure^4,8^. BSE is the recommended method in developing countries because it is easy, convenient, private, safe and requires no specific equipment^9^. Its purpose is to make women familiar with both the appearance and feel of their breasts as early as possible, so that they will be able to easily detect changes in their breast. Several studies have revealed that a positive association exists between the performance of BSE and detection of breast cancer, as most of the early breast tumor detections have been self-discovered^10,11^.

BSE for the early detection of breast cancer is not often done by many women. Studies^12,13^ affirm only 17% and 12% of women, respectively, were observed to perform BSE monthly. Such an observation has equally been noted among health personnel. For instance, a study conducted among nurses and midwives reported only 14% compliance^14^. As a result, most cases of breast cancer in women are diagnosed at an advanced stage due to late presentation. Although BSE is a simple, quick and cost-free procedure, it appears that many women either perform it incorrectly or not at all.

Early diagnosis of breast cancer has been clearly shown to reduce mortality and improve survival^8^. Combined results from randomized screening trials suggest that mammography reduces the risk of dying from breast cancer by 15–20%, and studies of modern mammography screening programs in Europe found that the risk of death from breast cancer was reduced by more than one-third^15^. Thus, knowledge and practice of breast cancer preventive measures and screening is therefore critical in the reduction of breast cancer morbidity and mortality.

However, poor awareness and knowledge about breast cancer symptoms and screening methods have been previously reported by several different studies^16^. To plan critically needed breast cancer awareness and education programs that optimally address Nigerian women civil servants, healthcare professionals and planners must know the women’s current level of understanding. Therefore, this cross-sectional study in State Secretariat, Ibadan, was performed to explore public knowledge of breast self-examination (BSE) and mammogram amongst women workers in Oyo State.

### Aims

The main aim of this study was to assess the level of knowledge and use of breast self-examination and mammogram among women of reproductive age in Oyo State Secretariat, Ibadan, Oyo State. Other objectives included: 1) Assess the level of knowledge of breast cancer, its risk factors and prevention measures; 2) Assess level of utilization of mammogram and breast self-examination; and 3) Identify the perceived barriers to the use of BSE and mammogram.

## METHODS

### Design

The study was a descriptive cross-sectional design that used a purposive sampling technique to select 204 consenting women from all specialities working in the State Secretariat, Agodi, Ibadan, to assess their views on breast cancer prevention actions (BSE and mammogram). Ethical approval was obtained from the Ethical Review Committee of the University of Ibadan, University College Hospital and from the Head of Service, Oyo State. With approval from the Head of Service, respondents’ consent was obtained, confidentiality of information was ensured and participants were not coerced to participate in the study and free to withdraw their participation at any stage of the study if they so desired without any negative consequences.

### Sample

The subjects for the study were women workers of reproductive age (20–62 years) in Oyo State Secretariat, Agodi, Ibadan. The total number of female workers in Oyo State Secretariat were 340 and the sample size for this study was determined using the Yamane formula^17^. The calculated sample size was 184. Adjusting the sample size for 10% attrition, brought it to 204. Purposive sampling technique was used to select all participants who met the eligibility criteria.

### Data collection

Data were collected using a structured validated close-ended questionnaire. The questionnaire consisted of five sections. Section A consisted of sociodemographic data. Section B asked questions on knowledge of breast cancer, its risk factors, signs and symptoms and preventive measures. Section C was on questions related to knowledge of BSE and mammogram. Section D was on level of utilization of mammogram and self-breast examination. Section E was concerned with barriers to the use of breast self-examination and mammogram.

The instrument was presented to research experts to ensure validity, while reliability was determined using a test-retest method among 10 female workers that were picked randomly from outside the study centre and yielded a coefficient of 0.75. The researchers administered the questionnaire to the respondents face-to-face and collected them after they were completed.

### Data analysis

Data obtained were coded and entered into a spread-sheet. Analysis was performed using the Statistical Package for Social Science, version 20. Descriptive statistics such as frequency counts, percentages, mean and standard deviation were used to summarize and present the results. Student’s t-test was used to compare the categorical variables at p<0.05. Logistics regression was used to identify awareness and level of utilization of breast self-examination and mammogram among female workers in State Secretariat, Oyo State, at 0.05 level significance.

## RESULTS

The 204 women who participated in the study had their questionnaires checked to ensure completion before retrieval. The return rate was 100%.

Of the respondents, 40.7% were in the age group 20–32 years (Table 1). Ethnic Yorubas represented the majority in the study consisting of 131 women (64.2%), while the least were 12 Hausas (5.9%). The majority (88.2%) had university education, while others (11.8%) were only diploma holders.

**Table 1:** Sociodemographic variables of the respondents

*VARIABLE*	*Frequency*	*Percentage (%)*
**Age group** (years)		
20-32	83	40.7
33-42	73	35.8
43-52	37	18.1
53-62	11	5.4
**Religion**		
Christianity	132	64.7
Islam	72	35.3
Traditional	-	0
**Educational qualification**		
OND/HND	24	11.8
Degree	109	53.4
Masters	71	34.8
**Ethnicity**		
Yoruba	131	64.2
Igbo	61	29.9
Hausa	12	5.9

### Knowledge on risk factors

Data on knowledge and risk of breast cancer revealed that 192 (94.1%) respondents heard about breast cancer, while 12 (5.9%), claimed they never heard of it. The sources of the 192 respondents’ information were: health workers (73, 38%), newspapers (71, 36.9%), parents (25, 13%), and social media (23, 12.1%). On the risk factors for breast cancer, 72 (37.5%) respondents reported genetics 23 (12.1%), diet 36 (18.6%), while others gave metaphysical reasons. On the symptoms, the majority 170 (88.6%) claimed a lump in the breast, change in breast shape 11 (5.7%) and shortness of breath. Genetic mutations were the most identified risk factor for breast cancer; 59 (30.7%) respondents claimed age, while 13 (6.8%) felt it resulted from post-menopausal hormone therapy.

The response of knowledge about the risk factors, causes, and symptoms of breast cancer showed that 17.2% (n=35) of the study population had poor knowledge of breast cancer and its risk factors. The cause, symptoms and risk factors of breast cancer were known by 82.8% (n=169) of the respondents. This means that the vast majority of women in the study had good knowledge of breast cancer. They were women who scored above the average of the total score, while those who scored below the average were women with poor knowledge (17.2%).

Table 2 gives the frequency and percentage distribution of respondents’ knowledge of BSE and mammogram and their significance in the prevention of breast cancer. The majority (66.2%) of the respondents agreed to go for breast cancer screening if they have a strong symptom, while the vast majority (94.1%) agreed to early screening being a good way of detecting breast cancer. Similarly, many (71.1%) knew that Magnetic Resonance Imaging is one method of screening for breast cancer. In the same way, many (77%) were aware of mammography and its usefulness in early detection of breast cancer.

**Table 2:** Frequency and percentage distribution of respondents’ knowledge of BSE and mammogram in the prevention of breast cancer (N=204)

*Statements*	*Yes*		*Don’t know*		*No*	

	*N*	*%*	*n*	*%*	*n*	*%*
Only people who have strong symptoms of breast cancer should undergo screening	135	66.2	-	0	69	33.8
I know that early screening is a good way of preventing breast cancer	192	94.1	-	0	12	5.9
Self-breast examination is a type of screening for breast cancer	108	52.9	84	41.2	12	5.9
Molecular Breast Imaging is a type of screening for breast cancer	122	59.8	70	34.3	12	5.9
Ultrasonography is a type of screening for breast cancer	157	77.0	35	17.2	12	5.9
Magnetic Resonance Imaging is a type of screening for breast cancer	145	71.1	47	23.0	12	5.9
I know about breast self-examination	179	87.7	13	6.4	12	5.9
Breast self-examination is an effective screening method for breast cancer which only takes five minutes to apply	107	52.5	72	35.3	25	12.3
Breast self-examination should be started at the age of 20 years	144	70.6	35	17.2	25	12.3
Breast self-examination can be done by the woman herself	157	77.0	35	17.2	12	5.9
Breast self-examination is a non-invasive procedure with no special material or tool requirements	181	88.7	23	11.3	-	0
I know about mammography	158	77.5	46	22.5	-	0
Mammography is useful in the early detection of breast cancer	155	76.0	49	24.0	-	0
Mammography uses low energy x-rays to examine human breast	96	47.1	95	46.6	13	6.4

Table 3 shows the level of utilization of the screening. More than half of the respondents (52.9%) did not practise breast self-examination at the age of 20 years, other (58.3%) respondents said they would never practise it. However, about half of the respondents were consistent with both the practice of BSE and mammogram, 47.1% and 58.8%, respectively.

**Table 3:** Frequency and percentage distributions of respondents’ level of utilization of mammogram and breast self-examination

*Statements*	*Yes*		*Don’t know*		*No*	

	*n*	*%*	*N*	*%*	*n*	*%*
I started practising breast self-examination at the age of 20 years	84	41.2	12	5.9	108	52.9
I have never had breast self-examination done	109	53.4	24	11.8	71	34.8
I presently practise breast self-examination	13	6.4	35	17.2	156	76.5
I practise every month breast self-examination	49	24.0	22	10.8	133	65.2
I will never practise breast self-examination	119	58.3	-	0	85	41.7
I am consistent with the practice of breast self-examination	97	47.5	11	5.4	96	47.1
I have never had mammogram done	36	17.6	25	12.3	143	70.1
I had mammogram done after I clocked 40 years	83	40.7	26	12.7	95	46.6
I do mammogram annually	120	58.8	11	5.4	73	35.8
I am consistent with the practice of mammogram	120	58.8	84	41.2	204	100.0

### Barriers and fears

On the barriers to using the mammogram and self-breast examination, 193 (94.6%) respondents maintained that breast cancer screening is time consuming. Fear of exposure to radiation discouraged 180 (88.2%) from doing mammography, while 122 (59.8%) claimed fear of the outcome of the mammogram and breast self-screening. Another 133 (65.2%) claimed they did not understand the process of breast self-examination and 168 (82.4%) suggested that they needed to know more about breast cancer screening.

Table 4 shows that there is significant association between the knowledge of breast cancer and the use of BSE and mammogram (p<0.05). It also shows significant association between level of education and the use of BSE and mammogram (p<0.05). Hence, the null hypothesis was rejected.

**Table 4:** Cross-tabulation of respondents’ knowledge, level of education, and use of BSE and mammogram

*Variable*		*Use of BSE and mammogram*		*Total*	*χ^2^*	*df*	*p*
		*Yes*	*No*				
**Knowledge**	Yes	35 41.2%	50 58.8%	85 100.0%	59.148	1	0.000
	No	0 0.0%	119 100.0%	119 100.0%			
	Total	35 17.2%	169 82.8%	204 100.0%			
**Level of education**	OND/HND	24 100.0%	0 0%	24	38.164	2	0.000
	Graduate	36 33.0%	73 67.0%	109			
	Masters	25 35.2%	46 64.8%	71			
	**Total**	85	119	204			

## DISCUSSION

The high level of knowledge demonstrated by the respondents is in agreement with studies in similar settings^18,19^, as well as in Turkey^20^. However, this level of knowledge is in contrast to other studies conducted in Turkey, where the majority of the women had little knowledge^21^, and among minorities in the United State^22,23^. Poor knowledge of the risk factors was also noted by studies in Nigeria^24^ and in the Middle East^25-27^.

Health workers and family members were the major sources of information on screening and knowledge of breast cancer according to researchers in similar settings^28-30^ while social media have also been identified as sources^31^. This view is also held by studies in developed countries^32,33^. However, information from family members and friends could be unreliable, thereby leading to fears and poor compliance. It is therefore important that oncology related health workers be trained in giving information to clients in various settings. This will be more correct, increase knowledge, improve use and reduce morbidity.

The fact that majority of the respondents were aware of the value of BSE in early detection of breast cancer and as a non-invasive procedure is in the right direction, in line with results from another study^34^, but in contrast with a study in a different setting^33^. Similarly, the participants’ knowledge of the value of mammography was supported by other studies. A Nigerian study noted that poor knowledge among their participants about mammography being an early detection measure^24^ was due to the fact that it was not readily available in a rural setting^34^. This could be attributed to the socioeconomic condition experienced in the countryside, especially among rural dwellers, where there is poor access, geographically and financially. Most of the rural dwellers are farmers with poor educational background that reduces their ability to access relevant information^31^. Also poor access roads prevent the sale of farm produce resulting in limited income, which further compounds the issue of knowledge about and travelling for a mammography. Improving accessibility to the rural areas could improve flow of information and increase knowledge of the value of a mammogram.

Findings from this study revealed low utilization of BSE and mammogram despite the high level of knowledge, in line with other studies^23,30^. However, an earlier study in the region found higher proportions of the practice of BSE but poor adherence to the process^28,32^. This could be attributed to increased awareness over the years, which led to more exposure but with no corresponding level of utilization. This suggests that the issue of wider utilization requires more than awareness and should be addressed by examining other sociocultural factors.

Among factors that constituted barriers were views of low risk and fear of having abnormal results; this has been corroborated by findings that views of not being at risk, fear, and anxiety of abnormal results are major barriers^35^. Also, the reported issue of not having enough time could be attributed to the long waiting experienced in most federal and state centers that offer screening services. The implication of this is that follow-up appointments should be streamlined in order to reduce waiting time and enhance compliance.

The issue of clinical and risk factors being significantly linked to respondents’ knowledge and educational level is in line with other studies^36,37^. A woman with a higher level of education is more likely to report promptly to the hospital for proper examination if any symptom suggestive of cancer is seen or felt; although earlier studies^37,39^ reported that educated women often forget to practise BSE.

### Implication to nursing and midwifery practice

There is a need to educate women on BSE and mammography on the significance of the practice and use of these methods for the early detection of breast cancer. Nurses and midwives should organize training sessions, especially on BSE, so that women can practise it on their own.

### Limitations

The research project aimed at assessing the knowledge and use of BSE and mammogram among women of reproductive age in Oyo State Secretariat, Ibadan, however the sample size constitutes only a small percentage of the civil servants in Nigeria, which limits the generalization of the study.

## CONCLUSIONS

Findings from this study demonstrate that although the majority of the respondents had adequate knowledge of BSE and mammogram, the level of utilization of these methods was still low. More so, a significant association was found between knowledge, level of education and use of BSE and mammogram. Identified barriers to wider utilization of the screening methods, were time, fears of the result of the screening, and other sociocultural factors, and these should be taken into account when designing interventions. This may reduce the barriers, facilitate wider utilization and ultimately lead to a decrease in the morbidity and mortality rate of breast cancer.

In as much as there are strategies in place for increasing awareness and sensitization about breast cancer screening, measures should also be in place to educate, motivate and encourage women to practise BSE and mammogram as preventive measures for the early detection of breast cancer.

Midwives should reinforce the need to practise these screening measures among women. Governments and other stake holders should provide support to facilitate the education of women and provide adequate information about preventive screening for breast cancer. Efforts should be directed to making mammogram more accessible, available and affordable for all, especially in rural areas.

## References

[1] GLOBOCAN (2012). Estimated Cancer Incidence, Mortality and Prevalence Worldwide.

[2] American Cancer Society (2015). Breast cancer facts and figures 2015-2016.

[3] Rahib L, Smith BD, Aizenberg R, Rosenzweig AB, Fleshman JM, Matrisian LM (2014). Projecting cancer incidence and deaths to 2030: the unexpected burden of thyroid, liver and pancreas in the United States. Cancer Research.

[4] American Cancer Society Breast cancer facts and figures 2014.

[5] Chleboski RT, Anderson GL, Grass M (2010). Estrogen plus progestin and breast cancer incidence and mortality in post menopausal women. JAMA.

[6] World Health Organisation Breast cancer: prevention and control.

[7] Leung J, McKenzie S, Martin J, Dobson A, McLaughlin D (2014). Effect of rurality on Screening for breast Cancer: a systematic review and Meta- analysis comparing mammography. Rural Remote Health;.

[8] Li J, Shao Z (2015). Mammography Screening in less developed Countries. Springerplus.

[9] Asuzu CC (2007). Knowledge, attitude and practice of self-breast examination among the female students of the University of Ibadan, Nigeria. Pakistan Journal of social Sciences.

[10] Nde FN, Assob JCN, Kwenti T, Njunda LD, Tainenbe TRD (2015). Knowledge, attitude and practice of breast self-examination among female undergraduate students in the University of Buea. BMC Res Notes.

[11] Sani AM, Yau SL (2018). Relationship between knowledge and practice of breast self-examination among female workers in Sokoto, Nigeria. Obstetrics and Gynecological International Journal.

[12] Nafissi N, Saghafinia M, Motamedi M (2012). A survey of breast cancer knowledge and attitude in Iranian women. Journal of Cancer research and therapy.

[13] Godazandeh G, Khani H, Khalilian AR (2008). Knowledge and practices related to breast cancer prevention in Iranian female population, multi-center study in 2004. Journal of Mazandaran University of Medical sciences.

[14] Majeda M, Kamel A, Samia S, Gamal M (2008). Knowledge and factors affecting breast self-examination among Kuwaiti women. Kuwait Medical J.

[15] US National library of Medicine Breast cancer screening, PDQ.

[16] Akhigbe and Omuemu (2009). Knowledge, attitude and practice of breast cancer screening among female health workers in a Nigerian urban city. BMC Cancer.

[17] Yamane T (1967). Statistics: An Introductory Analysis.

[18] Odusanya OO, Tayo OO (2001). Breast cancer knowledge attitudes and practice among nurses in Lagos, Nigeria. Acta oncol.

[19] Azubuike S, Okwuokei S (journal). Knowledge, attitude and practices of women towards breast cancer in Benin City, Nigeria. Annal of Medical health science research.

[20] Pinar ED, Dilek O, Beyhan O (2006). The knowledge and attitudes of breast self-examination and mammography in a group of women in a rural area in Western Turkey. BMC.

[21] Cetingoz R, Kentli S, Urok O, Demirtas EM, Eyiler F, Kinay M (2002). Turkish people’s knowledge of cancer and attitude towards prevention and treatment. J Cancer Educ.

[22] Liu L, Wang F, Li-Xiang Y, Zhi-Gang Y (2014). Breast cancer awareness among women in Eastern China: a cross-sectional study. BMC Public Health.

[23] Ko CM, Sadler G, Ryujin L, Dong A (2003). “Filipina American women’s breast cancer knowledge, attitudes and screening behaviors’. BMC Public Health.

[24] Oluwatosin OA, Oladepo O (2006). Knowledge of breast cancer and its early detection measures among rural women in Akinyele Local Government Area, Nigeria. BMC Cancer.

[25] El-Shinawi M, Youssef A, Alsara M, Mohamed KA, Mohamed MM (2013). Assessing level of breast cancer awareness among recently diagnosed patients in Ain Shams University Hospital. Breast.

[26] Radi SM (2013). Breast Cancer Awareness among Saudi Females in Jeddah. Asian Pacific Journal for Cancer Prevention.

[27] Alam A (2006). Knowledge of breast cancer and its risk and protective factors among women in Riyadh. Ann Saudi Med.

[28] Adebamowo CA, Ajayi OO (2000). Breast Cancer in Nigeria. West African Journal of Medicine.

[29] Ohaeri BM, Oladele EO, Ohaeri JU (2001). Social support needs and adjustment of cancer patients. East Journal of Medicine.

[30] Olowokere AE, Onibokun AC, Oluwatosin AO (2012). Breast cancer knowledge and screening practices among women in selected rural communities of Nigeria J. Public Health Epidemiol.

[31] Friedman LC, Moore A, Webb JA, Puryear LJ (1999). Breast cancer screening among ethnically diverse low-income women; in a general hospital psychiatry clinic. Gen Hosp Psychiatr.

[32] Jebbin NJ, Adotey JM (2004). Attitudes to knowledge and practice of breast self examination in Port Harcourt. Nig J Med.

[33] Jules CN, Tebit EK, Anna LN, Taddi RG (2014). Knowledge, attitude and practice of breast self-examination among female undergraduate students in the University of Baeu. BMC research notes.

[34] Aboserea M, Abdelgawad M, Wafik (2011). Early detection of breast cancer among females at fakous district Sharkia Governorate, Egypt. Life Science Journal.

[35] Maxwell AE, Bastani R, Warda US (2000). Demographic predictors of cancer screening among Filipino and Korean immigrants in the united states. Amj Prev Med.

[36] Maxwell CJ, Bancej CM, Snider J (2001). Predictors of mammography use among Canadian women aged 50-69: Findings from the 1996/97 National Population Health Surveys. CMAJ.

[37] Sensiba ME, Stewart DS (1995). Relationship of Perceived Barriers to Breast Self-Examination. Res Briefs.

[38] Austoker J (2003). Breast Self- Examination. BMJ.

